# Dietary factors associated with breast cancer among women in Ethiopia: a systematic review and meta-analysis of case–control studies

**DOI:** 10.3389/fnut.2025.1499634

**Published:** 2025-02-17

**Authors:** Habitamu Mekonen, Ayenew Negesse, Melese Linger Endalifer, Gebeyaw Molla, Zelalem Aneley

**Affiliations:** ^1^Department of Human Nutrition, College of Health Science, Debre Markos University, Debre Markos, Ethiopia; ^2^Department of Human Nutrition, Faculty of Chemical and Food Engineering, Bahir Dar Institute of Technology, Bahir Dar University, Bahir Dar, Ethiopia

**Keywords:** breast cancer, women, diet, risk factors, Ethiopia

## Abstract

**Background:**

Diet is the primary and largely modifiable factor associated with breast cancer risk. However, inconsistent findings were evidenced in many epidemiological studies and resulted in a lack of conclusiveness. Therefore, this systematic review and meta-analysis aimed to explore dietary risk factors that may predict breast cancer among Ethiopian women.

**Design, data source and eligibility criteria:**

A systematic review and meta-analysis was carried out. The articles were retrieved through electronic databases searching, including PubMed/Medline, Web of Science, Science Direct, EMBASE and Google Scholar. The Joanna Briggs Institute Meta-Analysis of Statistics Assessment and Review Instrument was applied for the critical appraisal. All case–control studies conducted in Ethiopia and reporting dietary factors of breast cancer in women were included in the final analysis.

**Data extraction:**

Two independent reviewers extracted the data using a standardized data extraction format in Excel software. Stata version 17 software was used for the data analysis. Cochran’s Q statistic with inverse variance (I^2^) was used to assess the presence of heterogeneity. A random effect model was used to estimate the odds ratio with a 95% confidence interval.

**Results:**

Overall, eight eligible articles with 2,774 women were included to explore the dietary predictors of breast cancer in Ethiopia. As a result, alcohol consumption (OR: 1.26, 95% CI: 1.01, 1.57), packed food intake (OR: 6.83; 95% CI: 4.56, 10.24), saturated fat/oil intake (OR: 1.51; 95% CI: 1.13, 2.02), meat consumption (OR: 6.08, 95% CI: 3.62, 10.22), and vegetable consumption (OR: 0.75, 95% CI: 0.49, 0.89) were identified as significant predictors of breast cancer among women in Ethiopia.

**Conclusion:**

The current study revealed a significant relationship between dietary factors and breast cancer. Avoiding the consumption of alcohol, saturated fats/oils, packed foods, and meat, coupled with promotion of vegetable consumption, could substantially contribute to reduce the burden of breast cancer among women in Ethiopia. Therefore, policymakers and other concerned bodies should provide routine community-based nutrition education to raise public awareness about the contribution of women’s dietary practices on their breast cancer risk.

## Background

Breast cancer (BC) is the most prevalent type of malignancy and the leading cause of women’s cancer-related deaths worldwide (1–3). It is a metastatic cancer characterized by abnormal breast cells and that divide uncontrolled, typically resulting in a lump or mass (4, 5). BC can commonly transfer to distant organs such as the bone, liver, lung, and brain, which mainly accounts for its incurability (5, 6). Therefore, early detection of this disease can result in a favorable prognosis and increased survival rates (7).

According to the American Cancer Society and International Agency for Research on Cancer (IARC) 2022 report, breast cancer represents 11.6% of all cancer cases and it constituted 23.8% of cancer cases among women (3). In Sub-Saharan African (SSA) countries, BC is the most prevalent cancer among women—where the key health system factors such as a shortage of human resources, challenges in accessing healthcare, diagnostic errors, poor management, and high treatment costs are exist (8, 9). More than half of the incidence and deaths of breast cancer occur in low-and middle-income countries (LMICs) (10). For instance, African countries had the highest age-standardized mortality rate (17.3 deaths per 100,000 annually) associated with breast cancer (11). In Ethiopia, breast cancer incidence is rising and accounts for 15,244 (22.6%) all cases of cancer and 8,159 (17%) cancer mortality annually (12). The Federal Ministry of Health of Ethiopia set strategic objectives for prevention and control of non-communicable disease (NCD) including breast cancer through minimizing risk factors and promoting a healthy lifestyles (13).

Multiple modifiable environmental factors, including an unbalanced diet, a lack of physical activity, a high body mass index (BMI), and high alcohol and/or tobacco consumption, are attributed to 90–95% breast cancer incidence (14, 15). Diet, the most important component of lifestyles, has a tremendous potential to influence the onset of breast cancer by altering the epigenome (16), and attributed to nearly 35% of all cancer cases (17). Excessive alcohol consumption, a high dietary fat intake, and overweight/obesity are among the modern lifestyle risk factors for breast cancer (17–19). For instance, according to global data on alcohol consumption and breast cancer incidence, alcohol intake is attributed for an estimated 144,000 new cases and 38,000 deaths of breast cancer per year, accounting for 8.6% of all incidence and 7.3% of mortality (18).

In contrast, extensive research reveals that dietary modifications could prevent approximately one-third of breast cancer cases (20). Many components of food have been identified as beneficial in reducing the risk of breast cancer subtypes (21, 22). For instance, adherence to the Mediterranean diet, mainly characterized by fruits, vegetables, fish and low-fat dairy, has shown positive results in decreasing the overall risk of onset of the malignancy (23, 24). Consuming a diet high in fruits and vegetables (FV) as part of a general healthy diet can help prevent several serious chronic diseases such as heart disease, type II diabetes, obesity, and a few cancers (25). However, there is no internationally accepted conclusion regarding the influence of dietary items on breast cancer (26, 27). Ethiopian cuisine and dietary habits include unique ingredients and preparation methods that could influence breast cancer risk. For instance, the use of certain spices, oils, or traditional foods may have protective or harmful effects that have not been adequately explored in our populations. Ethiopian women may have specific environmental exposures that would affect their dietary pattern and, consequently, their breast cancer risk. Besides, the socioeconomic status of women in Ethiopia may result in different access to healthcare and nutrition, which could influence dietary patterns and breast cancer risk. Thus, a comprehensive examination of these factors could provide insight into the particular dietary risk factors faced by Ethiopian women. Understanding the dietary determinants of breast cancer in specific populations, such as Ethiopian women, is critical for tailoring public health interventions and breast cancer prevention strategies that are culturally and contextually relevant. Thus, the identification of the associated dietary factors in which their modification could lead to reductions in breast cancer occurrence is a public health prioritized action area. Therefore, this review aimed to explore the contribution of dietary factors in the development of breast cancer among women in Ethiopia. The findings of this study can help health policymakers and health care providers to design locally appropriate and effective prevention and health promotion measures for the identified risk factors.

## Methods

### Searching strategies and tools

Electronic databases such as PubMed/Medline, Web of Science, Science Direct, Embase and Google Scholar were used to retrieve published articles related to determinants of breast cancer. This review was reported using the Preferred Reporting Items for Systematic Reviews and Meta-Analyses (PRISMA) guideline (28) (Supplementary file 1). Keywords or phrases including dietary factors, dietary pattern, dietary items, lifestyle factors, modifiable factors, dietary habits, nutritional factors, risk factors, ketogenic diet, breast cancer, breast neoplasm and Ethiopia with their respective Medical Subject Heading Terms (MeSH terms) combined by Boolean Operators (OR and AND) were used to retrieve relevant articles (Supplementary file 2). Unpublished research was searched using cross references and gray literature available in institutional libraries. The overall article searching was conducted through July 20, 2024.

### Eligibility criteria

Inclusion criteria: All case–control studies conducted in all regional sates and administrative cities of Ethiopia, and reporting dietary factors of breast cancers in women, regardless of pathological characteristics and stage of the tumor, were included in this meta-analysis. Besides, research that reported the current study outcome variable and solely published in English were included in the final analysis. Regarding publishing status, publication year, and sampling technique, no restriction was placed.

Exclusion criteria: Animal studies, case reports, conference abstracts, and studies with insufficient data for computing important variables were excluded in the final analysis. In addition, papers that were not fully accessible at the time of our search process were excluded after contacting the principal investigator via email at least two times.

### Studies selection

Two reviewers (HM and AN) had select articles by reading titles and abstracts. During the article selection process, the researchers were not aware of the decisions one another made. After this phase, two researchers (HM and ZA) independently read the full text of the articles. Articles that met the eligibility criteria were included in the systematic review. In cases where there was divergence between the researchers, the inclusion or exclusion of the articles was decided by consensus (HM, AN and ZA).

### Outcome variable and exposures

Outcome variable: The outcome variable of this study was breast cancer. Exposures: The main exposures of the current study were alcohol consumption (ever versus never), meat intake [including smoked, red, and processed meat] (yes versus no), saturated oil/fat consumption (yes versus no), packed food intake (yes versus no), fruit intake (yes versus no), and vegetable consumption (yes versus no).

Data extraction: Once, the studies which met the inclusion criteria were selected, two authors (HM and AN) independently extracted the data using a standardized data extraction format developed according to the 2014 Joanna Briggs Institute Reviewers’ Manual. From each primary study, the following data: author’s name, study area, study year, study design and technique, sample size, and odds ratio showing the impact of each exposure variable on breast cancer were extracted.

### Quality assessment

Prior to data extraction, the included studies were critically evaluated using the Joanna Briggs Institute Meta-Analysis of Statistics Assessment and Review Instrument (JBI-MAStARI) (29). The following JBI criteria were employed to evaluate the quality of case–control studies: 1.comparable groups; 2.cases and controls matched appropriately; 3.similar criteria used for identification of cases and controls; 4.valid and reliable measurement for exposure; 5.similar exposure measurement; 6.confounding factors identified; 7.strategies to deal with confounding factors; 8.outcomes assessed in valid and reliable way; 9.the exposure period of interest long enough; and 10.appropriate statistical analysis. Disagreements between the reviewers were settled through discussion. In the event that there were discrepancies between the two independent reviewers, a third reviewer was consulted. As a result, the final meta-analysis comprised the studies that scored ≥6 of the predefined quality measurement criteria.

We assessed and evaluated risk of bias in the studies that were selected using the 9-item rating scale developed by Hoy et al. (30). Sampling, data collection, reliability and validity of study tools, case definition, and prevalence periods were included in the tool. The rating scale categorized as low risk of bias (“yes” answers to domain questions) or high risk of bias (“no” answers to domain questions) for each articles. Each study was assigned a score of 1 (Yes) or 0 (No) for each domain, and these scores were summed to determine the level of risk of bias. Accordingly, scores of 7–9 were considered as having a “low risk of bias,” 5–6 a “moderate risk,” and 0–4 a “high risk.” For the final risk of bias classification, disagreements between the reviewers were resolved via consensus.

### Data analysis

First, the extracted data were computed in excel spread sheet and then imported to STATA version 17.0 for further analysis. Cochran’s Q statistic with inverse variance (I^2^) was used to assess the existence of statistical heterogeneity and to quantify it. Low, moderate and high heterogeneity were considered at 25, 50 and 75%, respectively (31). In addition, *p* value less than 0.05 was used to confirm the presence of heterogeneity across studies. As a result, statistically significant heterogeneity was detected (p value <0.05). Forest plot diagram was employed to present the association between breast cancer and exposure variables. To estimate the pooled impact of each exposure on breast cancer, a random effect meta-analysis (since heterogeneity was significant) using metan command was fitted and quantified through odds ratio with 95% confidence interval (95% CI). A meta-regression was computed to evaluate potential heterogeneity across studies.

## Results

### Primary studies selection process

**Figure 1:**
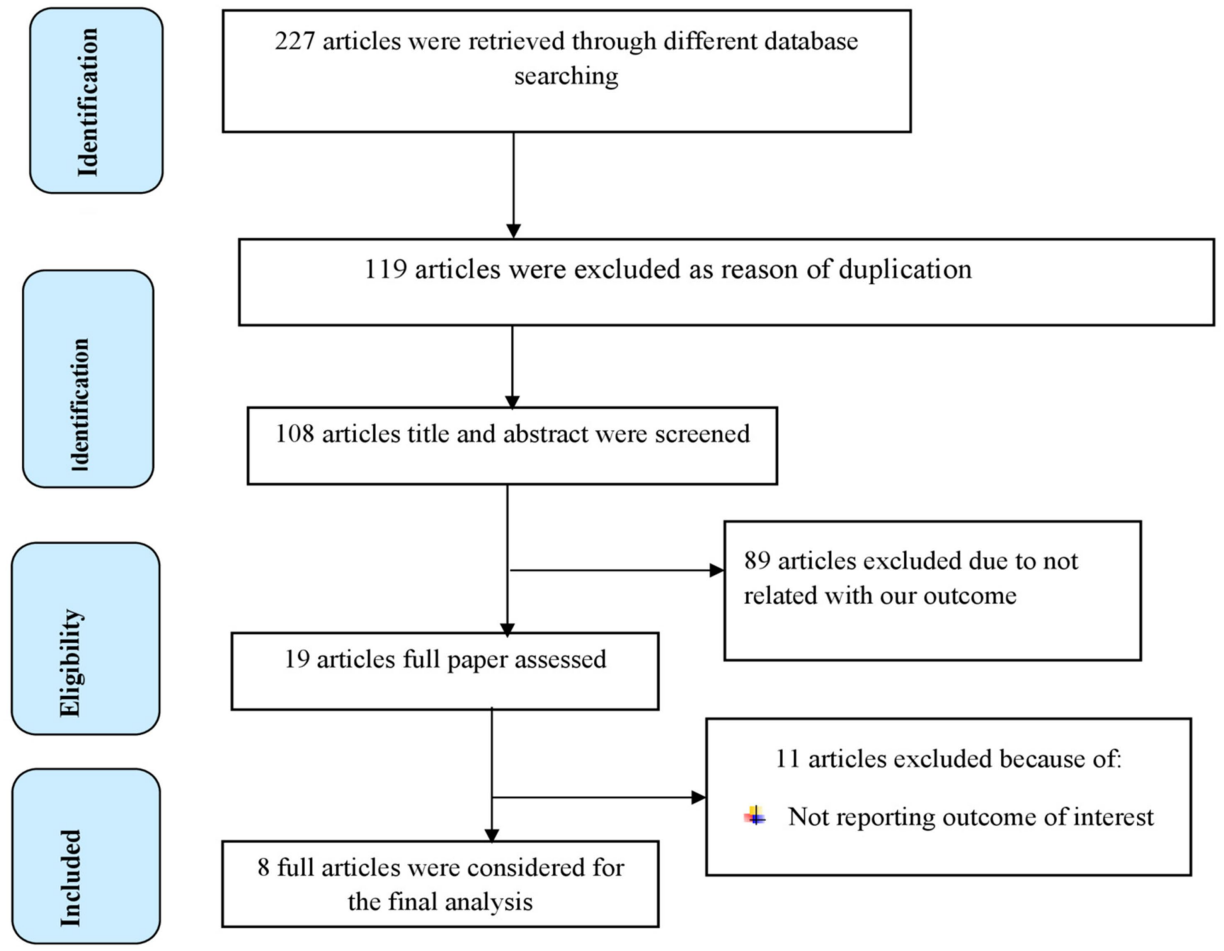
PRISMA flow diagram of included studies to evaluate the pooled effect of dietary factors on BC in women in Ethiopia.

A total of 227 articles were retrieved through searching from health and medical-related electronic data bases and other sources. Then 119 articles were excluded because of duplication. After screening the remaining 108 articles, 89 articles were excluded because they were irrelevant for the present study. The remaining 19 studies were assessed based on eligibility criteria, and 10 articles were removed because they did not report outcomes of interest. Finally, eight full articles had met the minimum eligibility criteria settled out by the JBI-MAStARI critical appraisal tool, and they were included in the final analysis to estimate the pooled effect of dietary factors on breast cancer in women in Ethiopia (Figure 1).

### Characteristics of the included studies

From included articles, 6 of them were published studies whereas the remaining two articles were unpublished. All of the included studies used a case control study design. A total of 2,774 reproductive-aged women were included in this meta-analysis. From the included studies, six studies from Addis Ababa (32–37), a country’s largest cancer diagnosis and treatment center, and the remaining two from Sidam region (38, 39). According to JBI-MAStARI critical appraisal tool, each included individual articles scored minimum of 6 to maximum of 8 out of 10 (Table 1). Concerning risk bias assessment based on a tool developed by Hoy et al., 6 (75%) studies had low risk of bias, and the remaining 2 (25%) studies had moderate risk of bias (Supplementary file 3). To see how studies with moderate risk of bias affected our estimates of pooled effect size, we computed it with exclusion of studies having a moderate risk of bias. However, the confidence intervals for pooled effect size with and without low methodological quality studies were overlapped, indicating no significant difference between them. These results suggest that the majority of the primary study authors have met high-quality standards. This gives credibility to our findings.

**Table 1:** Characteristics of the primary studies included in the meta-analysis of exploring the association between dietary factors and breast cancer among women in Ethiopia.

Author	Study year	Region	Study design	Sampling technique	Sample size (case: control ratio)	Exposures	JBI score
Tolessa et al.	2020	Addis Ababa	Case–control	Systematic	348 (1:2)	Breastfeeding, abortion, overweight/obesity, physical activity, OCs, meat intake, use of packed foods	8
Teshale et al.	2022	Sidama	Case–control	Convenient	388 (1: 2)	Breastfeeding, abortion, physical activity, OCs, alcohol use, fruit intake, vegetable intake, packed food intake, meat intake	7
Hassen et al.	2019	Addis Ababa	Case–control	Convenient	460 (1:1)	Overweight/obesity, vegetable intake, saturated fat/oils, meat intake, fruits intake, alcohol use	8
Letta et al.	2013	Addis Ababa	Case–control	Convenient	453 (1: 2)	Breastfeeding, overweight/obesity, alcohol use	6
Tseganesh et al.	2021	Addis Ababa	Case–control	Convenient	171(1: 2)	Breastfeeding, overweight/obesity, physical activity OCs, fruit intake, vegetable intake, meat intake, saturated fats/oils	7
Duche et al.	2017	Addis Ababa	Case–control	Convenient	220 (1: 1)	Breastfeeding, abortion, overweight/obesity, physical activity, OCs, vegetable intake, alcohol use, fruits intake	8
Kebede et al.	2023	Sidama	Case–control	Convenient	300 (1:3)	Breastfeeding, abortion, overweight/obesity, physical activity, OCs, saturated fats/oils, packed foods,	8
Mengesha et al.	2015	Addis Ababa	Case–control	–	434 (1: 1)	Breastfeeding, abortion, alcohol use	6

A meta-analysis of dietary factors of breast cancer among women in Ethiopia.

**Figure 2:**
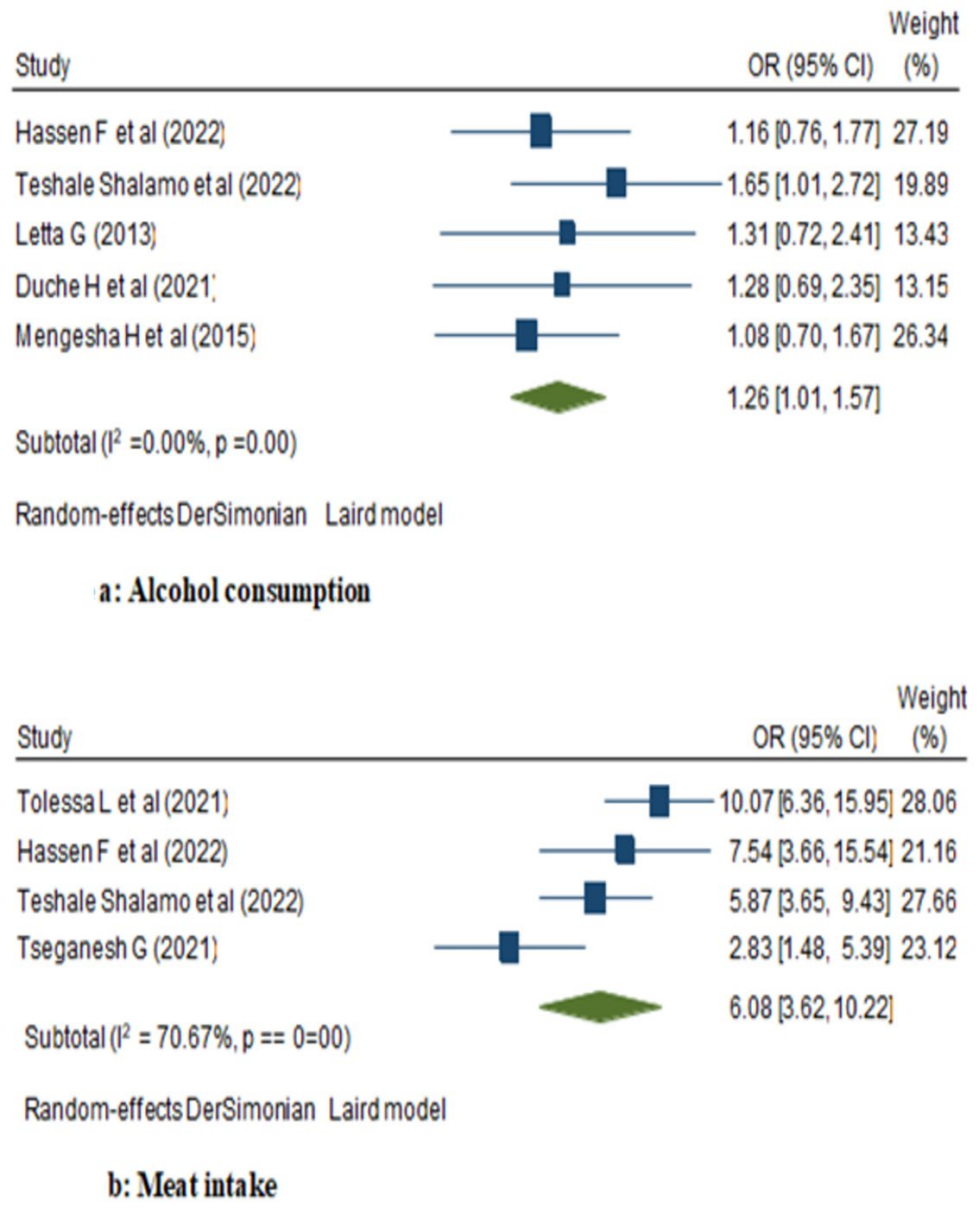
Forest plot showing the pooled effect of alcohol and meat consumption on breast cancer in women in Ethiopia.

**Figure 3:**
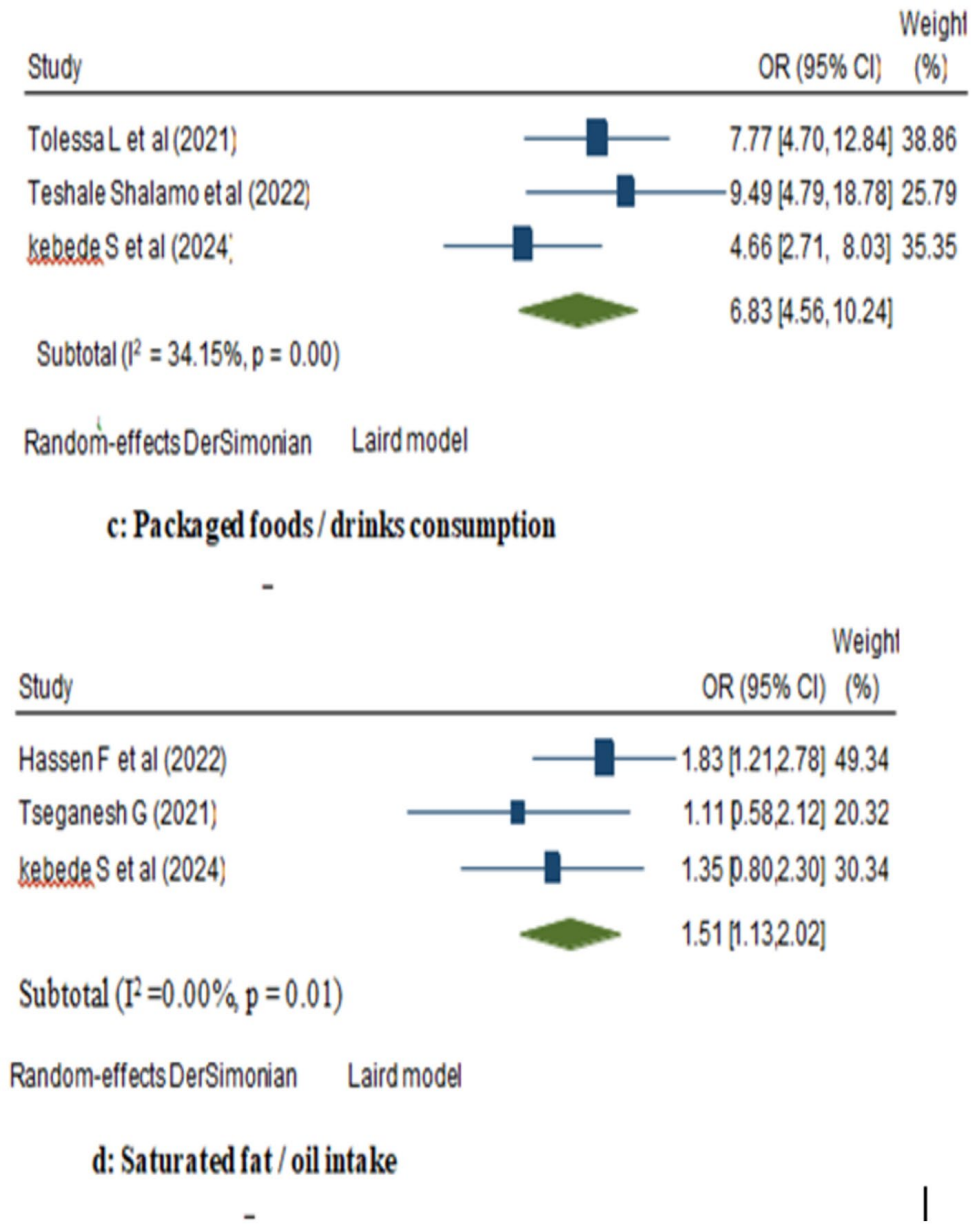
Forest plot showing the pooled effect of packaged foods and saturated fats/oils consumption on breast cancer in women in Ethiopia.

In the random model, the odds of breast cancer were 1.26 folds higher among women with ever alcohol use compared to those who never use alcohol (95% CI: 1.01, 1.57). Similarly, there was a favorable correlation between eating meat and a woman’s higher risk of breast cancer—where the likelihood to have breast cancer increased 6.08 folds (95% CI: 3.62, 10.22; Figure 2). Besides, women who consumed packaged foods or drinks had a 6.83-fold increased risk of breast cancer compared to those who did not (OR: 6.83; 95% CI: 4.56, 10.24). Moreover, among women, those who were users of saturated fats and oils had significantly higher odds of being breast cancer cases compared to those who did not (OR: 1.51; 95% CI: 1.13, 2.02; Figure 3).

**Figure 4:**
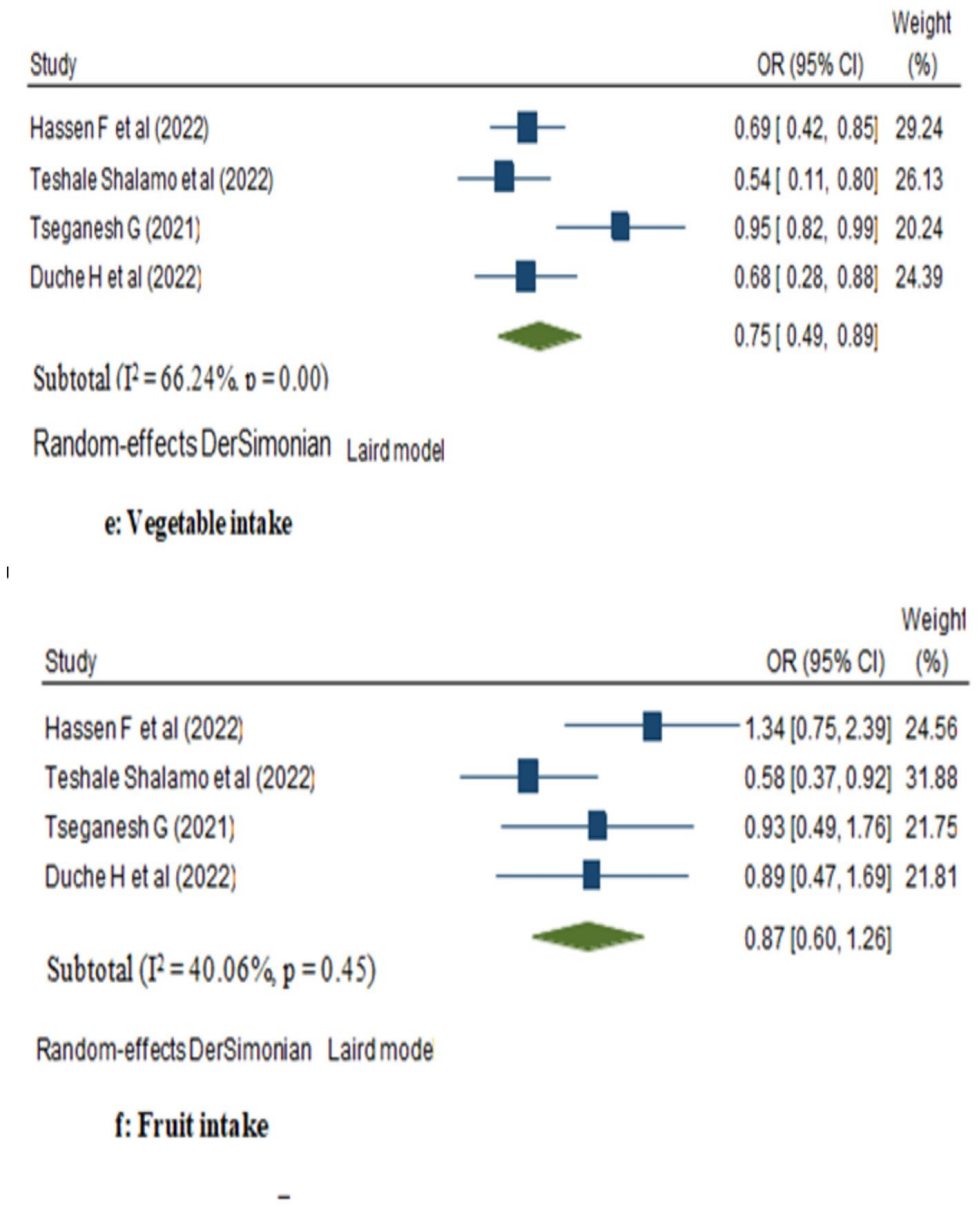
Forest plot showing the pooled impact of vegetables and fruits intake on breast cancer in women in Ethiopia.

This random model meta-analysis showed an inverse association between vegetables consumption and breast cancer risk. The odds ratio for women with consumption of vegetables is 0.75 (95% CI: 0.49, 0.89) compared with women who had not; vegetable consumption down the risk of women’s breast cancer risk by 25%. However, the likelihood of breast cancer was not statistically significant among women who had consumed fruits as compared to women who did not (OR: 0.87; 95% CI: 0.60, 1.26; Figure 4).

### Meta-regression

Regarding the effect of meat consumption on breast cancer in women, high heterogeneity (I^2^ = 70.67%, *p* value = 0.00) was observed across the included studies. To identify the potential sources of heterogeneity, meta-regression was carried out by considering sample size and study year, but none of these variables were found to be statistically significant (Table 2). Similarly, high heterogeneity (I^2^ = 66.24%, *p* value = 0.00) was observed across the included studies considered in exploring the association between vegetable consumption and breast cancer risk. To identify the potential sources of heterogeneity, meta-regression was carried out by considering sample size and study year, statistically insignificant was noted (Table 3).

**Table 2:** Meta-regression for the included studies to identify source of heterogeneity for the pooled effect of meat consumption on BC.

Variable	Coefficient	*p* value	95% CI	
Sample size	0.0018167	0.552	−0.0253539	0.0289873
Study year	−0.2019356	0.689	−5.033252	4.629381
_cons	407.8347	0.689	−9352.134	10167.8

**Table 3:** Meta-regression for the included studies to identify source of heterogeneity for the pooled effect of vegetable consumption on BC.

Variable	Coefficient	*p* value	95% CI	
Sample size	4.39e-07	1.000	−0.0182112	0.0182121
Study year	−0.3836043	0.569	−6.454128	5.686919
_cons	775.529	0.569	−11494.25	13045.31

## Discussion

The aim of the current systematic review and meta-analysis was to identify the dietary factors of BC in Ethiopia. It revealed that alcohol consumption, meat intake, packed food consumption, saturated oil/fat intake and vegetable intake were significant predictors of breast cancer. Evidence implies that, while risk reduction may be modest for any single factor evaluated in women, longer-term preventive dietary choices should reduce overall breast cancer risk over time.

Based on the current study, alcohol consumption was a risk factor for breast cancer among women. This result is consistent with other meta-analysis studies (40, 41). Alcohol is a group 1 human carcinogen linked to seven cancer types, including 40,000 new breast cancers in the European region in 2020. Alcohol-induced elevation of estrogen levels can lead to hormonal imbalance and contribute to carcinogenesis in women’s organs (6, 42). Acetaldehyde, the most toxic metabolite of alcohol, can induce DNA lesions and cell mutations, converting normal cells into cancerous ones (43). Furthermore, alcohol-related impaired nutrient intake may substantially increase the risk of breast cancer. However, many people are not aware of the risk of breast cancer from alcohol consumption (44). Therefore, it better find ways to replace alcohol with other beverages and adopt a global policy placed on alcohol.

Based on our findings, consumption of vegetables had a significant protective effect against breast cancer development. It was supplemented by other meta-analyses studies’ findings (45–47). Other meta-analyses evidenced that the intake of vegetable-fruit-soybean dietary patterns could lower the development of breast cancer (48). The anti-carcinogenic properties of vegetables are believed to result from their high levels of antioxidants, fatty acids, vitamins, and minerals. Antioxidant vitamins, fiber, and folate are thought to be useful elements in vegetables that protect DNA from oxidative damage by neutralizing reactive oxygen free radicals (45, 49). Furthermore, vegetables and fruits contain compounds with anti-inflammatory and detoxifying effects that positively influence metabolic processes and endothelial function (50, 51). Phytochemicals like phenolic acids and flavonoids, natural secondary plant metabolites in vegetables, are mostly responsible for a protective effect against abiotic or biotic stress and some cancer diseases through their ability to combat inflammation and oxidative stress by controlling interleukin (IL)-6 (52, 53). However, the present meta-analysis revealed no significant association between fruit intake and breast cancer. This finding of the current study was in line with a large European prospective study (54). In contrast, a population based cohort study conducted in the United States reported a statistically significant inverse relation between fruit intake and breast cancer risk (55). These inconsistent results across the studies could be due to differences in types of fruits consumed in the target populations, dietary measurement tools, and inequality regarding the consumption of such diets due to limited purchasing power of the population.

The current meta-analysis revealed that consumption of meat was associated with breast cancer in women. Similar findings also indicated that red meat consumption is directly associated with an increased risk of breast cancer (56, 57). Heterocyclic amines, n-nitroso compounds, and polycyclic aromatic hydrocarbons—all of which have the potential to cause cancer in humans—are present in red and processed meats (58). Furthermore, animal fats and saturated fats that are present in meat are linked with the increased risk of breast cancer, in particular of the ER+/ER− and HER2-subtypes (59, 60). A meta-analysis carried out by Farvid et al. to evaluate the effect of red and processed meat consumption on breast cancer also revealed that 6% of breast cancer risk was attributed to consumption of red and processed meat (61).

The increasing amount of packaged food consumption in modern diets begs important questions concerning its effects on public health, especially with regard to the risk of cancer. Breast cancer, one of the most prevalent cancers among women globally, has garnered significant attention in research in relation to dietary patterns. According to this study, packaged foods or drinks consumption had statistically significant favorable effect on breast cancer; women who have used packed foods or drinks were nearly 6.83 times more likely to have breast cancer risk compared to their counterparts. This result is consistent with studies from Iran that found consuming packaged foods and beverages, such as soft drinks and industrially produced juices, was linked to a significantly higher risk of breast cancer (62, 63). Packaged foods are mostly deficient in essential nutrients and dietary fiber and rich in sugar, bad fats, and sodium. These nutritional profiles have the potential to cause overweight and obesity, which are known risk factors for breast cancer, especially in postmenopausal women (64). Furthermore, many packaged foods have artificial ingredients, chemical additives, and preservatives like nitrates and nitrites with endocrine-disrupting properties, which could affect breast cancer risk through hormonal pathways (65). Highly processed foods such as packaged foods, instant soups, reconstituted meats, frozen meals, and shelf-stable snacks also contain substances that may significantly increase the overall risk for cancer and breast cancer (66).

The present meta-analysis also found a statistically significant association between saturated fat/oil intake and breast cancer risk. This result was consistent with studies conducted by Farvid et al. and Sabina et al., which indicated a positive association between higher intake of saturated fat and breast cancer risk (67, 68). Another study conducted in the United States also showed that eating saturated fat increases the risk of developing breast cancer (69). Another systematic review showed that an unhealthy high-fat diet may contribute to obesity and affect BC (70). The possible reason is that saturated fats could elevate low-density lipoprotein (LDL) cholesterol, influence adipose tissue, and generate free radicals, all of which may play a role in cancer progression (59). Additionally, a high-fat diet may lead to oxidative stress and the production of reactive oxygen species (ROS), which can damage DNA and alter gene expression, potentially activating oncogenes associated with cancer (71). In contrast, Boeke et al. reported insignificant associations between saturated fat intake and breast cancer risk (72). The possible reason for inconsistencies across studies might be due to biases in different dietary assessment tools and a lack of adjustment for the effect of total fat and carbohydrates that can interfere in estimating a true relationship between saturated fat and breast cancer risk.

Our study revealed that statistically significant changes in pooled estimate were not discovered with inclusion of studies with a moderate risk of bias. The possible reason might be the use of advanced statistical techniques, such as random-effects models, can account for variability between studies, resulting in solid pooled results even in the presence of lower-quality studies. This helps to limit the potential impact of any single study. Furthermore, studies with moderate risk of bias reporting similar results; their inclusion may not substantially alter the pooled estimate. Despite our efforts, we admit that bias may still influence our findings. Thus, future research should be performed in more diverse populations and with improved methodologies in order to demonstrate the validity of our findings.

### Strengths and limitations of the study

The combined sample size was large, with a relatively long follow-up period in many of these studies. The majority of the studies adjusted for important potential risk factors for breast cancer. Besides, a thorough searching using more comprehensive databases, study selection process and data extraction were performed correctly by two independent assessors. As a limitation of studies on the dietary risk factor of breast cancer, the included primary studies used dietary measurements, such as reliance on self-reported food intake data, which led to underestimation or overestimation of the true influence of dietary factors on breast cancer in women. While we attempt to synthesize a wide body of literature, including studies with a moderate risk of bias reduces the robustness of our findings. Finally, our study lacks prior registration.

## Conclusion

The current study revealed a significant relationship between dietary factors and breast cancer. Avoiding the consumption of alcohol, saturated fats/oils, packed foods, and meat, coupled with promotion of vegetable consumption, could substantially contribute to reduce the burden of breast cancer among women in Ethiopia. Thus, nutritional intervention should be considered as an integral part of the multimodal preventive strategy for BC. Therefore, policymakers and other concerned bodies should provide routine community-based nutrition education to raise public awareness about the contribution of women’s dietary practices on their breast cancer risk and promote consumption of vegetables—low-hanging vegetables of nutrition interventions. Ethiopia’s health system is organized into primary, secondary, and tertiary levels, which offer basic care, more comprehensive services, and specialized care. Although it has made significant strides in recent years, it still faces numerous challenges due to a shortage of resources. Therefore, Ethiopia Ministry of Health should made partnership with non-governmental organizations and international health bodies can provide additional resources and expertise in addressing health challenges. Finally, the current study recommend the future researchers to carry out experimental studies based on gene-diet interactions that may complement the current knowledge and would be an important step toward effective nutritional interventions to prevent breast cancer and improve its treatment.

## Data Availability

The raw data supporting the conclusions of this article will be made available by the authors, without undue reservation.
